# Establishing a standardized high touch cleaning (HTC) training and competency framework

**DOI:** 10.1017/ash.2025.90

**Published:** 2025-09-03

**Authors:** Joanna Yujun Tan, Glorijoy Shi En Tan, Darius Lian Lian Beh, Bee Fong Poh, Brenda Sze Peng Ang

**Affiliations:** 1 Department of Infection Prevention and Control (DIPC), Tan Tock Seng Hospital (TTSH), Singapore; 2 Department of Infectious Diseases, Tan Tock Seng Hospital, National Centre for Infectious Diseases (NCID), Singapore

**Keywords:** High touch cleaning, Patient environment, Training and Competency

## Abstract

**Objectives:** Environmental hygiene of patient zones in the wards of TTSH- a 1700 bedded hospital in Singapore is upkept through twice daily HTC. An outbreak in two wards end March 2023 with high levels of Adenosine Triphosphate (ATP) found on surfaces after cleaning corroborated that the cleaning process was ineffective ^[1]^. Though operatives undergo on-job training (OJT), they expressed difficulty in understanding the purpose of such cleaning and remembering the steps. To address these gaps, a new training and competency framework was developed. We thus sought to evaluate its usefulness in improving compliance to HTC. **Method:** The framework, effected from May 2023, consisted of three domains: standardized education, competency assessment, and feedback mechanisms^[2, 4]^. Educational materials explaining the importance of HTC and overall infection prevention were developed in three common languages to facilitate understanding for operatives of different races. Under the framework, all existing and new operatives undertake a 2-day classroom teaching and OJT, before a competency check. Upon passing the first competency, they are given a two-week probation. Another assessment is done before certifying them competent. Operatives who fail twice are redeployed to non-clinical areas. The audit team gave direct feedback during monthly audits to evaluate performance and provide ongoing support and reinforcement. **Results:** The compliance of HTC in the patient zone picked up immediately from 71% in April to 92% in June. However, a decrease to 68% was observed between September to December 2023, but soon picked up to 82% in February 2024 after retraining was conducted. Decrease in ATP levels after cleaning further validated increase efficiency of HTC. **Conclusions:** These results highlight that structured learning rapidly improves the thoroughness of cleaning ^[2,^ ^3]^. Ongoing assessment and feedback are essential to address subsequent deficiencies and for corrective actions to be taken promptly ^[2,^ ^4]^. This framework may be useful for teams seeking to optimize strategies in environmental hygiene.

**Acknowledgments:** The authors thank the frontline environmental services teams for conducting and providing results of the HTC audits.

